# Enhanced Adsorption of Epoxy‐Functional Nanoparticles onto Stainless Steel Significantly Reduces Friction in Tribological Studies

**DOI:** 10.1002/anie.202218397

**Published:** 2023-01-31

**Authors:** Csilla György, Paul M. Kirkman, Thomas J. Neal, Derek H. H. Chan, Megan Williams, Timothy Smith, David J. Growney, Steven P. Armes

**Affiliations:** ^1^ Dainton Building Department of Chemistry University of Sheffield Sheffield South Yorkshire S3 7HF UK; ^2^ Lubrizol Ltd. Hazelwood Derbyshire DE56 4AN UK

**Keywords:** Epoxy-Functional Nanoparticles, Polymerization-Induced Self-Assembly, Quartz Crystal Microbalance, RAFT Polymerization, Stainless Steel

## Abstract

Epoxy‐functional sterically‐stabilized diblock copolymer nanoparticles (ca. 27 nm) are prepared via RAFT dispersion polymerization in mineral oil. Nanoparticle adsorption onto stainless steel is examined using a quartz crystal microbalance. Incorporating epoxy groups within the steric stabilizer chains results in a two‐fold increase in the adsorbed amount, Γ, at 20 °C (7.6 mg m^−2^) compared to epoxy‐core functional nanoparticles (3.7 mg m^−2^) or non‐functional nanoparticles (3.8 mg m^−2^). A larger difference in Γ is observed at 40 °C; this suggests chemical adsorption of the nanoparticles rather than merely physical adsorption. A remarkable near five‐fold increase in Γ is observed for ca. 50 nm epoxy‐functional nanoparticles compared to non‐functional nanoparticles (31.3 vs. 6.4 mg m^−2^, respectively). Tribological studies confirm that chemical adsorption of the latter epoxy‐functional nanoparticles leads to a significant reduction in friction between 60 °C and 120 °C.

## Graphical abstract

Introduction of epoxy groups into sterically‐stabilized diblock copolymer nanoparticles leads to their efficient chemical adsorption onto stainless steel whereas the corresponding non‐functional nanoparticles merely undergo physical adsorption. Significantly greater adsorption occurs when the epoxy groups are placed within the steric stabilizer chains, which leads to enhanced lubrication in tribology studies.

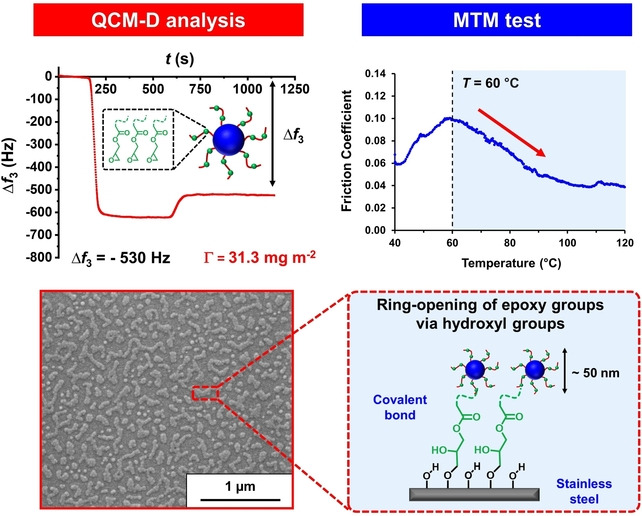

## Introduction

It is well‐documented that the addition of oil‐soluble polymers to automotive engine oils confers various benefits. For example, polyolefins or poly(*n*‐alkyl methacrylates) confer superior lubrication performance,[^1^, ^2^, ^3^, ^4^] polystyrene‐based diblock copolymers can act as effective diesel soot dispersants^[5]^ and star diblock copolymers can be used to control the viscosity‐temperature profile of engine oils.^[6]^

In 2010, Zheng and co‐workers reported that core‐crosslinked acrylic diblock copolymer nanoparticles reduced friction within the boundary lubrication regime as judged by mini‐traction machine (MTM) studies.^[7]^ Such nanoparticles were postulated to provide a physical barrier between the planar stainless steel surface and the stainless steel ball bearing that was placed in direct contact with it. However, these acrylic nanoparticles were prepared by a multi‐step process that involved protecting group chemistry and self‐assembly in dilute solution via solvent transfer from THF to mineral oil.^[7]^ Thus they are not readily amenable to industrial scale‐up. In contrast, in 2019 Derry et al. reported an efficient, scalable route for the preparation of methacrylic core‐crosslinked nanoparticles directly in mineral oil^[8]^ by the judicious combination of reversible addition‐fragmentation chain transfer (RAFT) polymerization[^9^, ^10^] with polymerization‐induced self‐assembly (PISA).[^11^, ^12^, ^13^, ^14^, ^15^, ^16^, ^17^, ^18^] Importantly, such sterically‐stabilized poly(stearyl methacrylate‐poly(benzyl methacrylate)‐poly(ethylene glycol dimethacrylate) (PSMA_31_‐PBzMA_200_‐PEGDMA_20_) nanoparticles also significantly reduced friction within the boundary lubrication regime in tribological experiments.^[8]^

Herein we demonstrate that introducing epoxy groups into similar methacrylic nanoparticles significantly enhances their adsorption from *n*‐dodecane onto planar stainless steel. This is achieved simply by statistical copolymerization of glycidyl methacrylate (GlyMA) with lauryl methacrylate (LMA) when preparing the steric stabilizer precursor block, prior to its chain extension using either BzMA or methyl methacrylate (MMA). Quartz crystal microbalance with dissipation (QCM‐D) and scanning electron microscopy (SEM) are used to compare the adsorption of such epoxy‐functional nanoparticles onto a model planar stainless steel substrate with that of (i) nanoparticles containing epoxy groups located within the cores and (ii)  nanoparticles bearing no epoxy groups. Finally, tribological experiments are conducted to assess whether nanoparticle adsorption leads to lower friction within the boundary lubrication regime.

## Results and Discussion

### Synthesis and Characterization of Spherical Nanoparticles

**Scheme 1:**
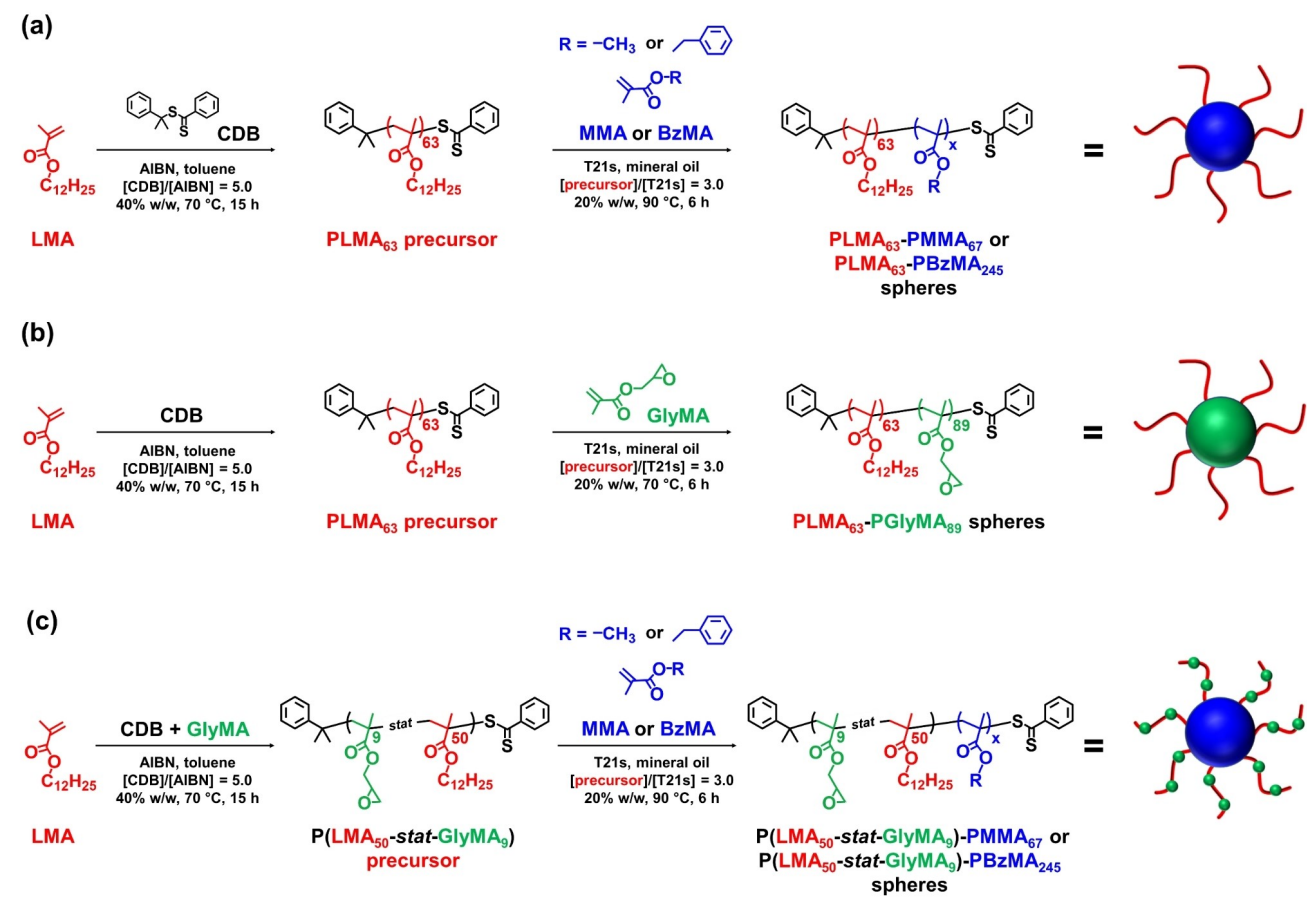
Synthesis of **(a)** poly(lauryl methacrylate)‐poly(methyl methacrylate) (PLMA_63_‐PMMA_67_) and poly(lauryl methacrylate)‐poly(benzyl methacrylate) (PLMA_63_‐PBzMA_245_), **(b)** poly(lauryl methacrylate)‐poly(glycidyl methacrylate) (PLMA_63_‐PGlyMA_89_), **(c)** poly(lauryl methacrylate‐*stat*‐glycidyl methacrylate)‐poly(methyl methacrylate) [P(LMA_50_‐*stat*‐GlyMA_9_)‐PMMA_67_] and poly(lauryl methacrylate‐*stat*‐glycidyl methacrylate)‐poly(benzyl methacrylate) [P(LMA_50_‐*stat*‐GlyMA_9_)‐PBzMA_245_] spherical nanoparticles via RAFT dispersion polymerization of methyl methacrylate (MMA), benzyl methacrylate (BzMA) or glycidyl methacrylate (GlyMA) in mineral oil at 20 % w/w solids.

RAFT dispersion polymerization in mineral oil was used to produce ca. 27 nm diameter epoxy‐functional PLMA_63_‐PGlyMA_89_ and P(LMA_50_‐*stat*‐GlyMA_9_)‐PMMA_67_ nanoparticles, as well as the corresponding non‐functional PLMA_63_‐PMMA_67_ nanoparticles (see Scheme 1). Similarly, both epoxy‐functional P(LMA_50_‐*stat*‐GlyMA_9_)‐PBzMA_245_ and non‐functional PLMA_63_‐PBzMA_245_ spheres of approximately 50 nm diameter were also prepared in mineral oil (see Scheme 1). In each case, a high final monomer conversion (≥95 %) was achieved within 6 h. THF GPC analysis indicated comparable *M*_n_ values for similar copolymer compositions, as shown in Figure S1. A narrow molecular weight distribution (*M*_w_/*M*_n_≤1.18) and a high blocking efficiency was obtained for each synthesis (see Figure S1).

**Figure 1:**
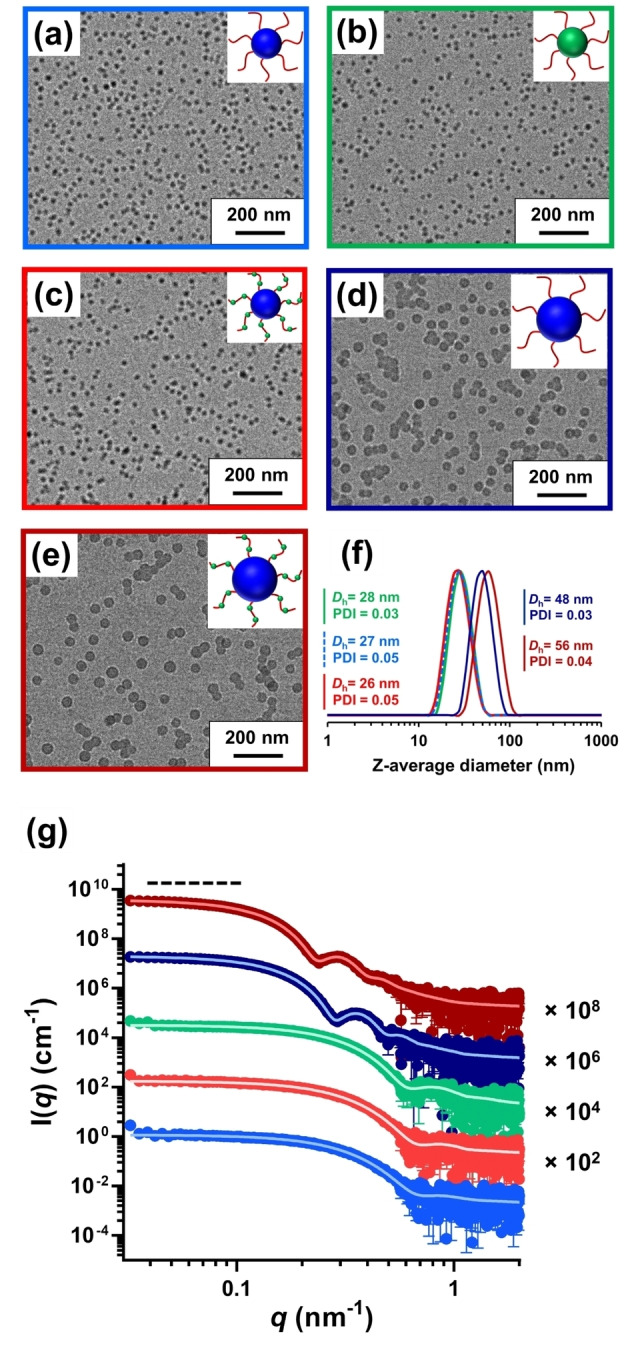
Representative TEM images obtained for **(a)** PLMA_63_‐PMMA_67_ nanoparticles (light blue frame), **(b)** PLMA_63_‐PGlyMA_89_ nanoparticles (green frame), **(c)** P(LMA_50_‐*stat*‐GlyMA_9_)‐PMMA_67_ nanoparticles (red frame), **(d)** PLMA_63_‐PBzMA_245_ nanoparticles (dark blue frame) and **(e)** P(LMA_50_‐*stat*‐GlyMA_9_)‐PBzMA_245_ nanoparticles (dark red frame) prepared at 20 % w/w solids in mineral oil. **(f)** DLS data recorded for 0.1 % w/w dispersion of PLMA_63_‐PMMA_67_ nanoparticles (light blue dashed trace), PLMA_63_‐PGlyMA_89_ nanoparticles (green trace), P(LMA_50_‐*stat*‐GlyMA_9_)‐PMMA_67_ nanoparticles (red trace), PLMA_63_‐PBzMA_245_ nanoparticles (dark blue trace) and P(LMA_50_‐*stat*‐GlyMA_9_)‐PBzMA_245_ nanoparticles (dark red trace). **(g)** SAXS patterns and corresponding data fits (solid white lines) recorded for 1.0 % w/w dispersions of PLMA_63_‐PMMA_67_ nanoparticles (light blue data), PLMA_63_‐PGlyMA_89_ nanoparticles (green data), P(LMA_50_‐*stat*‐GlyMA_9_)‐PMMA_67_ nanoparticles (red data), PLMA_63_‐PBzMA_245_ nanoparticles (dark blue data) and P(LMA_50_‐*stat*‐GlyMA_9_)‐PBzMA_245_ nanoparticles (dark red data; originally prepared at 20 % w/w solids in mineral oil) at 20 °C. Dashed line is for guidance to the eye and indicates a low *q* gradient of zero in each case.

The spherical morphology was confirmed in each case via TEM analysis (see Figure 1a–e). Particle size distributions were assessed by DLS and SAXS. The former technique indicated a hydrodynamic diameter (*D*_h_) of 27 nm with a polydispersity index (PDI) of 0.05 for PLMA_63_‐PMMA_67_ nanoparticles, 28 nm (PDI=0.03) for PLMA_63_‐PGlyMA_89_ nanoparticles, and 26 nm (PDI=0.05) for P(LMA_50_‐*stat*‐GlyMA_9_)‐PMMA_67_ nanoparticles (see Figure 1f). A *D*_h_ of 48 nm (PDI=0.03) was determined for PLMA_63_‐PBzMA_245_ nanoparticles while the P(LMA_50_‐*stat*‐GlyMA_9_)‐PBzMA_245_ nanoparticles had a *D*_h_ of 56 nm (PDI=0.04), see Figure 1f. SAXS is a powerful characterization technique that can provide detailed information regarding a wide range of polymer colloids.[^18^, ^19^, ^20^, ^21^] SAXS patterns recorded for PLMA_63_‐PMMA_67,_ PLMA_63_‐PGlyMA_89_, P(LMA_50_‐*stat*‐GlyMA_9_)‐PMMA_67_, PLMA_63_‐PBzMA_245_ and P(LMA_50_‐*stat*‐GlyMA_9_)‐PBzMA_245_ nanoparticles are shown in Figure 1g. It is well‐known that the nanoparticle morphology can be inferred from the low *q* gradient of an *I*(*q*) vs. *q* plot.^[22]^ For each nanoparticle formulation, a low *q* gradient of zero was observed (see Figure 1g), which is consistent with a spherical morphology. Fitting such patterns using a spherical micelle model^[23]^ provides the volume‐average particle diameter and the mean aggregation number, *N*_agg_. Overall volume‐average diameters of 21.6±0.4 nm (*N*_agg_=140), 23.0±0.2 nm (*N*_agg_=130) and 21.6±0.4 nm (*N*_agg_=150) were determined for the PLMA_63_‐PMMA_67_, PLMA_63_‐PGlyMA_89_ and P(LMA_50_‐*stat*‐GlyMA_9_)‐PMMA_67_ nanoparticles, respectively. Satisfactory fits to SAXS patterns recorded for the PLMA_63_‐PBzMA_245_ and P(LMA_50_‐*stat*‐GlyMA_9_)‐PBzMA_245_ nanoparticles resulted in volume‐average diameters of 40.6±2.7 nm (*N*_agg_=390) and 46.8±3.9 nm (*N*_agg_=620), respectively. In all cases, these diameters are slightly lower than the corresponding *D*_h_ values determined by DLS (see Table S1). This difference is not unexpected since DLS reports a z‐average diameter, so this technique should always oversize relative to SAXS.

### QCM‐D Studies of Nanoparticle Adsorption onto a Planar Stainless Steel Substrate

QCM‐D has been used by many research groups to study the physical adsorption of nanoparticles from aqueous solution onto various model planar substrates.[^24^, ^25^, ^26^, ^27^, ^28^, ^29^, ^30^]

There are also various QCM‐D studies of the adsorption of small‐molecule surfactants, such as glyceryl monooleate (GMO),^[31]^ fatty amines (e.g., octadecylamine, *N*‐tallowalkyl‐1,3‐propanediamine)[^31^, ^32^] or fatty acids (e.g., stearic‐, oleic‐ or linoleic acids)[^33^, ^34^] onto stainless steel, iron oxide or silica substrates from *n*‐alkanes. Such compounds are friction‐reducing agents,[^35^, ^36^] which is believed to be a direct result of their enhanced adsorption onto metal surfaces via the chelate effect, hydrogen bonding or van der Waals interactions.[^31^, ^36^, ^37^, ^38^] Recently, Gmür et al. used QCM‐D to study the adsorption of a series of poly(lauryl methacrylate)‐based diblock copolymers onto a planar iron oxide substrate from hexadecane. Such copolymers were prepared by RAFT solution polymerization using pentafluorophenyl methacrylate as reactive repeat units to enable introduction of nitrodopamine functional groups. The resulting anchor block led to a relatively high adsorbed mass in the form of a *brush‐like* surface layer and excellent friction reduction in tribological experiments.^[3]^ Similar observations were reported by the same team for poly(lauryl methacrylate)‐based diblock copolymers bearing a carboxylic acid‐based anchoring block.^[4]^ Herein we report the first QCM‐D study of the adsorption of epoxy‐functional diblock copolymer *nanoparticles* onto planar stainless steel from a non‐polar solvent (*n*‐dodecane).

**Figure 2:**
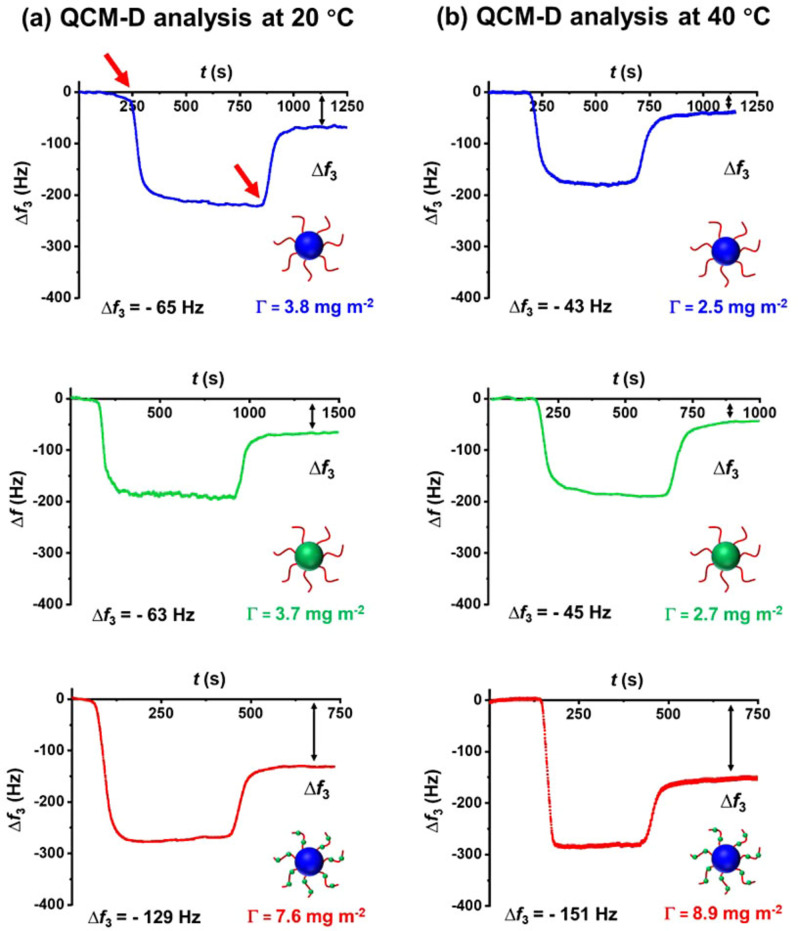
QCM‐D analysis showing the change in frequency (Δ*f*_3_) observed during the adsorption of PLMA_63_‐PMMA_67_ nanoparticles (blue data), PLMA_63_‐PGlyMA_89_ nanoparticles (green data) or P(LMA_50_‐*stat*‐GlyMA_9_)‐PMMA_67_ nanoparticles (red data) in turn from 1.0 % w/w copolymer dispersions in *n*‐dodecane onto a stainless steel substrate at a flow rate of 0.5 mL min^−1^: **(a)** at 20 °C and **(b)** at 40 °C. In part **(a)**, the first red arrow indicates when the nanoparticle dispersion was first introduced into the QCM cell while the second arrow corresponds to the rinsing step with pure *n*‐dodecane. Each curve is shown for a single measurement but good reproducibility was observed for duplicate experiments. Black double‐headed arrows indicate the final change in frequency (Δ*f*_3_) in each case, from which the adsorbed mass can be calculated.

First, adsorption of the ca. 27 nm epoxy‐functional PLMA_63_‐PGlyMA_89_ nanoparticles, epoxy‐functional P(LMA_50_‐*stat*‐GlyMA_9_)‐PMMA_67_ nanoparticles and non‐functional PLMA_63_‐PMMA_67_ nanoparticles were compared via QCM‐D experiments performed at 20 °C. Initially, pure *n*‐dodecane was introduced into the QCM‐D cell to obtain a baseline (see Figure 2), followed by switching to a 1.0 % w/w nanoparticle dispersion. Nanoparticle adsorption occurred at this stage (see Figure 2a, first red arrow) and subsequently returning to a flow of pure *n*‐dodecane enabled removal of weakly adsorbed nanoparticles from the surface (see Figure 2a, second red arrow). Typical dissipation data obtained during adsorption of the P(LMA_50_‐*stat*‐GlyMA_9_)‐PMMA_67_ nanoparticles is shown in Figure S2.

According to Figure 2a, employing the P(LMA_50_‐*stat*‐GlyMA_9_)‐PMMA_67_ nanoparticles resulted in a significantly greater change in frequency (Δ*f*_3_=−129 Hz) relative to that recorded for either the PLMA_63_‐PGlyMA_89_ nanoparticles (Δ*f*_3_=−63 Hz) or the non‐functional PLMA_63_‐PMMA_67_ nanoparticles (Δ*f*_3_=−65 Hz). In each case, the Δ*f*_3_ data provided in Figure 2 were average values obtained from two consistent measurements (see Table S2). The adsorbed mass per unit area can be calculated from Δ*f*_3_ (see Figure 2, black double‐headed arrows) using the Sauerbrey equation:^[39]^

(1)

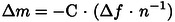




where C is a mass sensitivity constant, which is equal to 0.177 (mg (m^2^ Hz)^−1^) for a 5 MHz crystal,^[40]^ and *n* is the overtone number. To determine the final adsorbed mass of nanoparticles in mg m^−2^, we chose to use the third harmonic (*n*=3) of the resonance frequency. This is because the fundamental frequency (*n*=1) is rather sensitive to experimental artifacts.[^41^, ^42^]

It is emphasized that the Sauerbrey model assumes a rigid adsorbed layer and hence neglects viscoelastic effects. If the layer is appreciably viscoelastic, employing the Sauerbrey equation leads to an underestimate of the mass of the adsorbed layer.[^29^, ^43^] Monitoring the dissipation (*D*) in real time provides useful information regarding the viscoelasticity of the adsorbed nanoparticle layer.[^44^, ^45^] In principle, the adsorbed layer can be assumed to be rigid provided that the change in dissipation (Δ*D*) is relatively small compared to Δ*f*.[^46^, ^47^] Furthermore, Reviakine et al. reported that the Sauerbrey equation can be safely used if Δ*D*_n_/(−Δ*f*_n_/n)≪4×10^−7^ Hz^−1^ for a 5 MHz sensor.[^43^, ^47^] As shown in Table S2, all Δ*D*_3_/(−Δ*f*_3_/3) data were well below this threshold value. Thus the Sauerbrey equation should be valid for the nanoparticle adsorption experiments performed herein. Accordingly, adsorbed amounts of 7.6, 3.7 and 3.8 mg m^−2^ were calculated for the P(LMA_50_‐*stat*‐GlyMA_9_)‐PMMA_67_, PLMA_63_‐PGlyMA_89_ and PLMA_63_‐PMMA_67_ nanoparticles, respectively. Thus, introducing epoxy groups into the steric stabilizer block resulted in a two‐fold increase in the adsorbed mass per unit area compared to the non‐functional PLMA_63_‐PMMA_67_ nanoparticles. However, introducing almost ten times more epoxy groups into the nanoparticle *cores* produced no discernible increase in the adsorbed amount relative to these reference nanoparticles. Hence the precise spatial location of the epoxy groups within the nanoparticles is of critical importance for achieving their enhanced adsorption onto stainless steel. Moreover, our preliminary experiments (data not shown) suggest that placing a *single* epoxy group at the end of each steric stabilizer chain using an epoxy‐functional RAFT agent^[48]^ is not sufficient to promote greater nanoparticle adsorption. Thus it seems that *multiple* epoxy groups per copolymer chain are required to achieve this objective. In future work, it would be interesting to establish the *optimum* number of epoxy groups per copolymer chain that are required for *maximum* nanoparticle adsorption.

To estimate the corresponding fractional surface coverage (*Θ*) in each case, the QCM‐D adsorbed amount (Γ) was compared to the theoretical adsorbed mass (Γ_t_) estimated for a fully‐coated substrate of unit surface area. Γ_t_ was calculated using Equation 2:
(2)

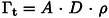




where *A* is equal to 1 m^2^, *D* is the overall nanoparticle diameter (ca. 27 nm in this case) and *ρ* is the density of either the PGlyMA core (*ρ*∼1.07 g cm^−3^ at 20 °C) or the PMMA core (*ρ*=1.18 g cm^−3^ at 20 °C). Accordingly, a *Θ* of 0.24 was determined for P(LMA_50_‐*stat*‐GlyMA_9_)‐PMMA_67_ nanoparticles, 0.13 for PLMA_63_‐PGlyMA_89_ nanoparticles and 0.12 for PLMA_63_‐PMMA_67_ nanoparticles.

There are two possible explanations for the significantly higher surface coverage obtained for the epoxy‐functional P(LMA_50_‐*stat*‐GlyMA_9_)‐PMMA_67_ nanoparticles. In principle, the surface hydroxyl (i.e., Fe‐OH) groups on the stainless steel substrate can simply form hydrogen bonds with the polar epoxy groups. Alternatively, such surface hydroxyl groups can ring‐open the epoxy groups to form covalent bonds. It is important to note that physical adsorption via hydrogen bonding should be less favorable at higher temperature. In contrast, such conditions should promote chemical adsorption.

To distinguish between these two adsorption mechanisms, the QCM‐D experiments were repeated at 40 °C, which is the upper limit temperature for our instrument set‐up (see Figure 2b). This higher temperature led to a modest but discernible *increase* in adsorbed amount for the P(LMA_50_‐*stat*‐GlyMA_9_)‐PMMA_67_ nanoparticles (Γ=8.9 mg m^−2^; *Θ*=0.28), whereas a significant *reduction* was observed for both the PLMA_63_‐PGlyMA_89_ nanoparticles (Γ=2.7 mg m^−2^; *Θ*=0.09) and the PLMA_63_‐PMMA_67_ nanoparticles (Γ=2.5 mg m^−2^; *Θ*=0.08). Thus these experiments suggest that a chemical reaction between the epoxy and hydroxyl groups occurs when employing the P(LMA_50_‐*stat*‐GlyMA_9_)‐PMMA_67_ nanoparticles, whereas the other two types of nanoparticles merely undergo physical adsorption (most likely via hydrogen bonding interactions between the methacrylic ester groups on the PLMA chains and the Fe‐OH groups present at the surface of the stainless steel). This is consistent with our recent observation that higher temperature enhances the ring‐opening of epoxy groups by water.^[49]^ Moreover, both Hatton et al. and Docherty et al. reported that hydroxyl groups can ring‐open epoxy groups via nucleophile substitution even at ambient temperature.[^50^, ^51^] Furthermore, Xu and co‐workers recently used sum‐frequency generation (SFG) vibrational spectroscopy to study the chemical reaction between epoxy rings and surface Al‐OH groups when curing an epoxy adhesive on sapphire.^[52]^ Moreover, the same team reported enhanced adhesion for an epoxy‐amine adhesive when cured in contact with a stainless steel substrate. Again, direct spectroscopic evidence was provided for a chemical reaction between the epoxy rings and the Fe‐OH groups.^[53]^

**Figure 3:**
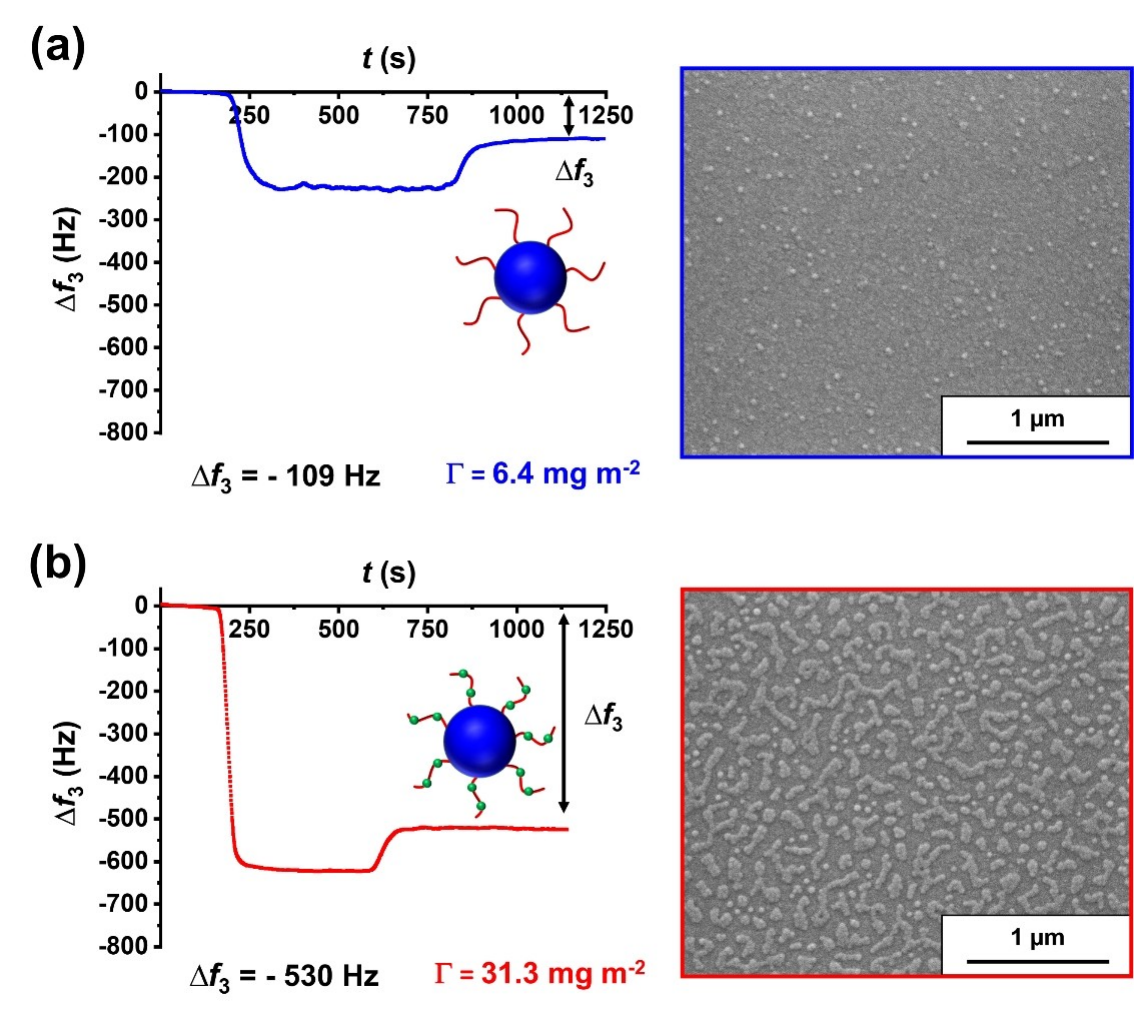
QCM‐D data obtained for the adsorption of **(a)** PLMA_63_‐PBzMA_245_ nanoparticles and **(b)** P(LMA_50_‐*stat*‐GlyMA_9_)‐PBzMA_245_ nanoparticles in turn from 1.0 % w/w copolymer dispersions in *n*‐dodecane onto a stainless steel substrate at a flow rate of 0.50 mL min^−1^ at 20 °C. Each curve is shown for a single measurement but good reproducibility was observed for duplicate experiments. The corresponding SEM images recorded for each nanoparticle‐coated stainless steel substrate after these QCM‐D experiments are also shown. The black double‐headed arrows indicate the final change in frequency (Δ*f*_3_).

Next, the adsorption of ca. 50 nm diameter epoxy‐functional P(LMA_50_‐*stat*‐GlyMA_9_)‐PBzMA_245_ nanoparticles onto stainless steel was compared to that obtained for non‐functional PLMA_63_‐PBzMA_245_ nanoparticles of similar size. To produce larger spheres in mineral oil, PBzMA was chosen as a core‐forming block instead of PMMA. This is because targeting higher PMMA DPs leads to the formation of colloidally unstable aggregates.^[54]^ As shown in Figure 3, increasing the particle size only resulted in a modest increase in adsorbed amount (Γ=6.4 mg m^−2^) when using the PLMA_63_‐PBzMA_245_ nanoparticles. In contrast, an approximate *five‐fold* increase in adsorbed amount (Γ=31.3 mg m^−2^) was obtained for the P(LMA_50_‐*stat*‐GlyMA_9_)‐PBzMA_245_ nanoparticles (see Table S3). Using Equation 2, assuming an overall nanoparticle diameter of 50 nm and taking the density of PBzMA to be 1.179 g cm^−3^, fractional surface coverages of 0.11 and 0.53 were calculated for the non‐functional and epoxy‐functional nanoparticles, respectively.

This remarkable difference is consistent with the observations made for the ca. 27 nm nanoparticles. Moreover, it indicates that nanoparticle adsorption is independent of the chemical nature of the core‐forming block (i.e., PMMA vs. PBzMA). Furthermore, the ca. 50 nm diameter nanoparticles are sufficiently large to be imaged via SEM after their adsorption onto stainless steel (see Figure 3). Such SEM images can be used to estimate surface coverage (*Θ*) using *ImageJ* software.

This approach enabled *Θ* values of ca. 0.10 and ca. 0.50 to be calculated for PLMA_63_‐PBzMA_245_ and P(LMA_50_‐*stat*‐GlyMA_9_)‐PBzMA_245_ nanoparticles, respectively. These fractional coverages are comparable to the corresponding *Θ* data determined from QCM‐D studies (see Table S3).

### Tribology Experiments Using 50 nm Nanoparticles

Previously, Zheng et al.^[7]^ and Derry et al.^[8]^ reported excellent lubrication performance for acrylic and methacrylic core‐crosslinked diblock copolymer nanoparticles, respectively. In these prior studies, Stribeck curves suggested a dramatic reduction in the friction coefficient at low entrainment speeds (i.e. within the boundary lubrication regime) compared to glycerol monooleate (GMO), which is a well‐known friction‐reducing agent. Zheng et al.^[7]^ postulated that this was the result of elastic deformation of the nanoparticles once they diffused within the asperity contact area.[^7^, ^8^] Herein, we sought to determine whether there is a direct relation between lubrication performance and nanoparticle adsorption. Accordingly, we compared the lubrication performance of the ca. 50 nm epoxy‐functional P(LMA_50_‐*stat*‐GlyMA_9_)‐PBzMA_245_ nanoparticles with that of the non‐functional PLMA_63_‐PBzMA_245_ nanoparticles.

Initially, Stribeck curves were recorded for 2.5 % w/w nanoparticle dispersions at 40, 60 and 80 °C while reducing the entrainment speed (*v*_e_) from 3000 to 30 mm s^−1^ using a slide‐to‐roll ratio (SRR) of 50 % under a constant applied load of 37 N (see Figure S3). At 40 °C, very similar data were obtained for the two nanoparticle dispersions (see Figure S3c). Moreover, these experiments indicate that a *v*_e_ of approximately 200 mm s^−1^ lies within the so‐called ‘mixed lubrication’ regime at this temperature. This intermediate regime lies between the hydrodynamic (full fluid film) and boundary (metal‐on‐metal contact) lubrication regimes.^[55]^ In principle, lowering the entrainment speed reduces the film thickness created by the oil between the metal surfaces, which should lead a higher number of asperity contacts and hence a higher friction coefficient (see Figure S3c).

Since the QCM‐D data suggests enhanced adsorption for the epoxy‐functional nanoparticles at higher temperature (see Figure 2), we examined more demanding MTM test conditions. Thus, the entrainment speed was fixed at 200 mm s^−1^ and a slide‐to‐roll ratio (SRR) of 50 % was employed under an applied load of 37 N while heating from 40 to 120 °C. In principle, the viscosity of the nanoparticle dispersion should be lower at higher temperature, which means that the onset of the “mixed lubrication” regime should occur at a higher entrainment speed (see Figure S3a–b). Hence additional asperity contacts and a higher friction coefficient were expected at elevated temperature.

**Figure 4:**
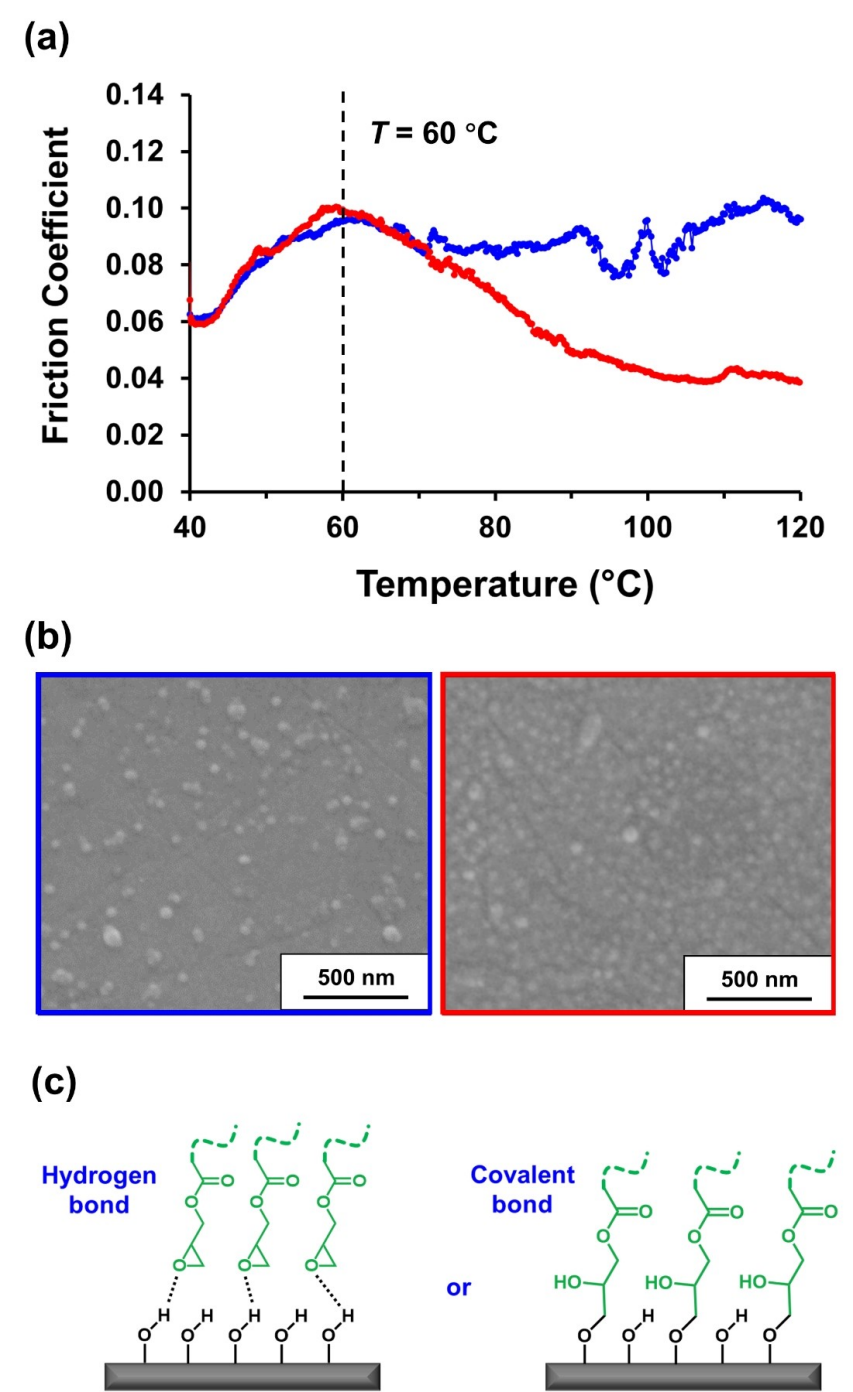
**(a)** Friction coefficient *vs*. temperature data obtained for a 2.5 % w/w dispersion of PLMA_63_‐PBzMA_245_ nanoparticles (blue data) and epoxy‐functional P(LMA_50_‐*stat*‐GlyMA_9_)‐PBzMA_245_ nanoparticles (red data). Data were recorded at an entrainment speed of 200 mm s^−1^ with a slide‐to‐roll ratio (SRR) of 50 % under an applied load of 37 N. **(b)** SEM images recorded for the MTM disks following these tribology experiments when using the PLMA_63_‐PBzMA_245_ nanoparticles (blue frame) and the P(LMA_50_‐*stat*‐GlyMA_9_)‐PBzMA_245_ nanoparticles (red frame), respectively. The epoxy groups in the latter nanoparticles clearly promote much stronger adsorption, which correlates with the significant reduction in friction coefficient observed above 60 °C. **(c)** Two possible mechanisms for the enhanced adsorption of epoxy‐functional P(LMA_50_‐*stat*‐GlyMA_9_)‐PBzMA_245_ nanoparticles onto stainless steel: (i) hydrogen bond formation between the polar epoxy rings and the surface hydroxyl groups (i.e., Fe‐OH) or (ii) epoxy ring‐opening reaction with the same surface hydroxyl groups to produce covalent bonds. The experimental evidence reported herein supports the latter interaction, for which there is also some literature precedent.[^52^, ^53^]

For the 40–60 °C regime, the two sets of data are essentially identical. A gradual increase in friction coefficient is observed for both the epoxy‐functional P(LMA_50_‐*stat*‐GlyMA_9_)‐PBzMA_245_ nanoparticles and the non‐functional PLMA_63_‐PBzMA_245_ nanoparticles (see Figure 4a). Above 60 °C (which corresponds to the boundary lubrication regime at a *v*_e_ of 200 mm s^−1^; see Stribeck curves shown in Figure S3a–b), a significant reduction in the friction coefficient from 0.09 to 0.04 was observed for the epoxy‐functional nanoparticles, whereas there is almost no change in this parameter for the non‐functional nanoparticles. This suggests that higher temperatures lead to more epoxy‐functional nanoparticles becoming located within the asperity contact area, despite the relatively harsh experimental conditions. Indeed, inspecting the surface of the MTM disks (within the test area) after these tribology experiments by SEM revealed a significant difference in the nanoparticle surface coverage, see Figure 4b.

Digital image analysis using *ImageJ* software indicated a relatively high fractional surface coverage of 0.45 for the epoxy‐functional P(LMA_50_‐*stat*‐GlyMA_9_)‐PBzMA_245_ nanoparticles but only 0.09 for the corresponding non‐functional nanoparticles. In the former case, it is perhaps worth emphasizing that the chemically‐adsorbed nanoparticles remain intact despite the relatively harsh conditions to which they were subjected during the tribological experiments. These MTM studies and SEM observations are consistent with our QCM‐D findings and provide further (albeit indirect) evidence for the likely chemical adsorption of the nanoparticles onto stainless steel, see Figure 4c.

## Conclusion

The adsorption of ca. 27 nm epoxy‐functional P(LMA_50_‐*stat*‐GlyMA_9_)‐PMMA_67,_ epoxy‐functional PLMA_63_‐PGlyMA_89_ and non‐functional PLMA_63_‐PMMA_67_ nanoparticles onto stainless steel from *n*‐dodecane was assessed using QCM‐D at 20 °C. The highest adsorbed amount was obtained when epoxy groups were incorporated within the steric stabilizer chains, rather than being located within the nanoparticle cores. Conducting these QCM‐D experiments at 40 °C led to a significantly higher adsorbed amount for the epoxy‐functional P(LMA_50_‐*stat*‐GlyMA_9_)‐PMMA_67_ nanoparticles. For purely physical adsorption, a lower adsorbed amount should be obtained under such conditions. Thus, such temperature‐dependent studies suggest that nanoparticle adsorption involves a chemical reaction between the epoxy groups and the Fe‐OH groups located at the stainless steel surface. Indeed, there is some recent literature precedent for such surface chemistry.[^52^, ^53^] Increasing the nanoparticle diameter from ca. 27 nm to ca. 50 nm resulted in an approximate five‐fold increase in the adsorbed amount for P(LMA_50_‐*stat*‐GlyMA_9_)‐PBzMA_245_ nanoparticles compared to that for the non‐functional PLMA_63_‐PBzMA_245_ nanoparticles, with the corresponding QCM‐D fractional surface coverages estimated to be 0.53 and 0.11, respectively. Furthermore, replacing the core‐forming PMMA block with PBzMA confirmed that the enhanced adsorption of such epoxy‐functional spheres is independent of the nature of the core‐forming block.

The ca. 50 nm diameter nanoparticles were then examined for tribology experiments using an MTM set‐up. When heating from 60 °C to 120 °C at a constant entrainment speed and slide‐to‐roll ratio, the epoxy‐functional P(LMA_50_‐*stat*‐GlyMA_9_)‐PBzMA_245_ nanoparticles reduced the friction coefficient dramatically compared to the corresponding non‐functional PLMA_63_‐PBzMA_245_ nanoparticles. In this case, *postmortem* SEM studies indicated a significantly higher surface coverage for the adsorbed epoxy‐functional nanoparticles. Such studies provide strong evidence for chemical adsorption of such nanoparticles via an epoxy‐hydroxyl ring‐opening reaction at elevated temperature.

Finally, given their relative ease of synthesis directly in mineral oil using potentially scalable chemistry, such epoxy‐functional nanoparticles appear to offer important advantages compared to other polymer‐based lubricating systems reported in the literature such as polymer brushes.[^56^, ^57^]

## Conflict of interest

The authors declare no conflict of interest.

1

## Supporting information

As a service to our authors and readers, this journal provides supporting information supplied by the authors. Such materials are peer reviewed and may be re‐organized for online delivery, but are not copy‐edited or typeset. Technical support issues arising from supporting information (other than missing files) should be addressed to the authors.

Supporting Information

## Data Availability

The data that support the findings of this study are available in the supplementary material of this article.
