# Supplementary material for: Enhanced Adsorption of Epoxy‐Functional Nanoparticles onto Stainless Steel Significantly Reduces Friction in Tribological Studies
Source: Angew Chem Int Ed Engl. 2023 Jan 31;62(10):e202218397. doi: 10.1002/anie.202218397 (PMC10962596; doi:10.1002/anie.202218397)
Supplement: Supplementary file 1 — Supporting Information [file ANIE-62-0-s001.pdf]

Angewandte Chemie
Eine Zeitschrift der Gesellschaft Deutscher Chemiker

Supporting Information

Enhanced Adsorption of Epoxy-Functional Nanoparticles onto Stainless Steel Significantly Reduces Friction in Tribological Studies

*C. György, P. M. Kirkman, T. J. Neal, D. H. H. Chan, M. Williams, T. Smith, D. J. Growney, S. P. Armes**

Supporting Information

Table of Contents

Experimental S2

Figure S1. GPC curves recorded for the $PLMA_{63}$ and P(LMA_{50}-*stat*-$GlyMA_{9}$) precursors and the diblock copolymers S9

Table S1. Summary table for SAXS fitting parameters and DLS data S10

Figure S2. Dissipation data recorded during the adsorption of P(LMA_{50}-*stat*-$GlyMA_{9}$)-$PMMA_{67}$ nanoparticles S10

Table S2. Summary table of Δf_3, ΔD_3, $\Delta D_3/(-\Delta f_3/3)$, Γ, Γ_t and Θ data obtained for the ~27 nm nanoparticles S11

Table S3. Summary table of Δf_3, ΔD_3, $\Delta D_3/(-\Delta f_3/3)$, Γ, Γ_t and Θ data obtained for the ~50 nm nanoparticles S11

Figure S3. Stribeck curves obtained for $PLMA_{63}$-$PBzMA_{245}$ and P(LMA_{50}-*stat*-$GlyMA_{9}$)-$PBzMA_{245}$ nanoparticles S12

References S13

Experimental

Materials

Methyl methacrylate (MMA, 99%) was purchased from Alfa Aesar (Germany), passed through basic alumina to remove its inhibitor and then stored at –20 °C prior to use. Lauryl methacrylate (LMA, 96%), glycidyl methacrylate (GlyMA, 97%), benzyl methacrylate (BzMA, 96%) cumyl dithiobenzoate (CDB), $CDCl_3$ and *n*-dodecane were purchased from Merck (UK) and used as received. 2,2′-Azobisisobutyronitrile (AIBN) was obtained from Molekula (UK) and *tert*-butyl peroxy-2-ethylhexanoate (T21s) was purchased from AkzoNobel (The Netherlands). CD_2Cl_2 was purchased from Goss Scientific (UK). Tetrahydrofuran was obtained from VWR Chemicals (UK). Methanol and toluene were purchased from Fisher Scientific (UK). Group III hydroisomerized mineral oil (viscosity = 4.3 cSt at 100 °C) was kindly provided by Lubrizol Ltd. (Hazelwood, Derbyshire, UK).

Synthesis of poly(lauryl methacrylate) ($PLMA_{63}$) precursor *via* RAFT solution polymerization in toluene

A $PLMA_{63}$ precursor was synthesized at 40% w/w solids according to a previously reported synthesis protocol.[1] Briefly, this synthesis was conducted as follows. LMA (77.0 g; 302.8 mmol), CDB (1.50 g; 5.5 mmol; target DP = 55), AIBN (181 mg; 1.10 mmol; CDB/AIBN molar ratio = 5.0) and anhydrous toluene (118.1 g) were weighed into a 250 mL round-bottomed flask. This reaction mixture was purged with a stream of nitrogen gas for 30 min at 20 °C and the sealed flask immersed in a preheated oil bath at 70 °C. The reaction solution was stirred continuously and the ensuing polymerization was quenched after 15 h by exposing the reaction solution to air while cooling the flask to 20 °C. A final LMA conversion of 90% was determined by ^{1}H NMR spectroscopy. The crude polymer was purified by three consecutive precipitations into a ten-fold excess of methanol (with redissolution in THF after each precipitation). The mean DP of this PLMA precursor was calculated to be 63 using ^{1}H NMR spectroscopy by comparing the ten aromatic protons assigned to the cumyl and dithiobenzoate end-groups at 7.10-8.00 ppm to the two oxymethylene protons attributed to PLMA at 3.75–4.20 ppm. THF GPC analysis using a refractive index detector and a series of near-

monodisperse poly(methyl methacrylate) calibration standards indicated an M_n of 12 600 g mol^{-1} and an M_w/M_n of 1.19.

Synthesis of poly(lauryl methacrylate-*stat*-glycidyl methacrylate) [P(LMA$_{50}$-*stat*-GlyMA$_9$)] precursor *via* RAFT solution polymerization in toluene

The P(LMA$_{50}$-*stat*-GlyMA$_9$) precursor was synthesized at 40% w/w solids using a recently reported synthesis protocol.[1] Briefly, LMA (63.0 g; 247.8 mmol), GlyMA (5.5 g; 38.5 mmol), CDB (1.50 g; 5.50 mmol; target DP = 52), AIBN (181 mg; 1.10 mmol; CDB/AIBN molar ratio = 5.0) and anhydrous toluene (105.3 g) were weighed into a 250 mL round-bottomed flask. Anhydrous toluene was used to minimize the potential loss of epoxy groups *via* ring-opening with water. This reaction mixture was purged with nitrogen for 30 min and the sealed flask was immersed in a preheated oil bath at 70 °C. The reaction solution was stirred continuously and the ensuing polymerization was quenched after 15 h by exposing the reaction solution to air while cooling the flask to 20 °C. An overall comonomer conversion of 90% was determined by ^{1}H NMR spectroscopy. The crude copolymer was purified by three consecutive precipitations into a ten-fold excess of methanol (with redissolution in THF after each precipitation). The overall mean DP of this P(LMA-*stat*-GlyMA) precursor was calculated to be 59 (i.e., 50 LMA units and 9 GlyMA units per copolymer chain), by using ^{1}H NMR spectroscopy to compare the ten aromatic protons assigned to the cumyl and dithiobenzoate end-groups at 7.10-8.00 ppm to the two oxymethylene protons attributed to the LMA repeat units at 3.85–4.20 ppm and the methine proton corresponding to the epoxide ring at 3.10-3.30 ppm, respectively. THF GPC analysis using a refractive index detector and a series of near-monodisperse poly(methyl methacrylate) calibration standards indicated an M_n of 12 300 g mol^{-1} and an M_w/M_n of 1.19.

Synthesis of poly(lauryl methacrylate)-poly(glycidyl methacrylate) [PLMA$_{63}$-PGlyMA$_{89}$] spherical nanoparticles *via* RAFT dispersion polymerization of GlyMA in mineral oil

PLMA$_{63}$-PGlyMA$_{89}$ spherical nanoparticles were prepared using a recently reported synthesis protocol.[1] Briefly, PLMA$_{63}$-PGlyMA$_{89}$ spherical nanoparticles were synthesized at 20% w/w solids using the following protocol. PLMA$_{63}$ precursor (1.50 g; 92.0 µmol), GlyMA (1.18 g; 8.28 mmol; target DP = 90), T21s initiator (6.64 mg;

30.7 µmol; precursor/T21s molar ratio = 3.0; 10.0% v/v in mineral oil) and mineral oil (10.74 g) were weighed into a glass vial and this reaction mixture was purged with nitrogen for 30 min. The sealed vial was immersed in a preheated oil bath at 70 °C and the reaction mixture was magnetically stirred for 6 h. ^{1}H NMR analysis indicated 99% GlyMA conversion by comparing the integrated vinyl signal at 6.17 ppm assigned to GlyMA monomer to the integrated epoxy methine signals corresponding to both PGlyMA and GlyMA at 3.20–3.33 ppm. THF GPC analysis using a refractive index detector and a series of near-monodisperse poly(methyl methacrylate) calibration standards indicated an M_n of 22 000 g mol^{-1} and an M_w/M_n of 1.18. A hydrodynamic diameter (D_h) of 28 nm (polydispersity index = 0.03) was determined by DLS.

Synthesis of poly(lauryl methacrylate-*stat*-glycidyl methacrylate)-poly(methyl methacrylate) [P(LMA_{50}-*stat*-$GlyMA_9$)-$PMMA_{67}$] spherical nanoparticles *via* RAFT dispersion polymerization of MMA in mineral oil

P(LMA_{50}-*stat*-$GlyMA_9$)-$PMMA_{67}$ spherical nanoparticles were prepared by following a recently reported synthesis protocol.[1] Briefly, P(LMA_{50}-*stat*-$GlyMA_9$)-$PMMA_{67}$ spherical nanoparticles were synthesized at 20% w/w solids as follows. P(LMA_{50}-*stat*-$GlyMA_9$) precursor (2.00 g; 140.13 µmol), T21s initiator (10.10 mg; 46.71 µmol; precursor/T21s molar ratio = 3.0; 10.0% v/v in mineral oil) and mineral oil (11.97 g) were weighed into a glass vial and purged with nitrogen for 30 min. MMA monomer (1.04 mL; 9.81 mmol; target DP = 70) was degassed separately then added to the reaction mixture *via* syringe. The sealed vial was immersed in a preheated oil bath at 90 °C and the reaction mixture was magnetically stirred for 6 h. ^{1}H NMR analysis indicated 95% MMA conversion by comparing the integrated methyl signal at 3.77 ppm assigned to MMA monomer to the integrated methyl signal corresponding to PMMA at 3.55–3.72 ppm. THF GPC analysis using a refractive index detector and a series of near-monodisperse poly(methyl methacrylate) calibration standards indicated an M_n of 21 100 g mol^{-1} and an M_w/M_n of 1.17. A hydrodynamic diameter (D_h) of 26 nm (polydispersity index = 0.05) was determined by DLS.

Synthesis of poly(lauryl methacrylate)-poly(methyl methacrylate) [$PLMA_{63}$-$PMMA_{67}$] spherical nanoparticles *via* RAFT dispersion polymerization of MMA in mineral oil

$PLMA_{63}$-$PMMA_{67}$ spherical nanoparticles were synthesized at 20% w/w solids as follows. $PLMA_{63}$ precursor (1.40 g; 85.89 µmol), T21s initiator (6.19 mg; 28.63 µmol; precursor/T21s molar ratio = 3.0; 10.0% v/v in mineral oil) and mineral oil (8.04 g) were weighed into a glass vial and purged with nitrogen for 30 min. MMA monomer (0.64 mL; 6.01 mmol; target DP = 70) was degassed separately, then added to the reaction mixture *via* syringe. The sealed vial was immersed in a preheated oil bath at 90 °C and the reaction mixture was magnetically stirred for 6 h. ^{1}H NMR analysis indicated 95% MMA conversion by comparing the integrated methyl signal of the monomer at 3.76 ppm to the integrated methyl signal of the polymer at 3.55–3.70 ppm. THF GPC analysis indicated an M_n of 21 200 g mol^{-1} and an M_w/M_n of 1.14. A hydrodynamic diameter (D_h) of 27 nm (polydispersity index = 0.05) was determined by DLS.

Synthesis of poly(lauryl methacrylate)-poly(benzyl methacrylate) [$PLMA_{63}$-$PBzMA_{245}$] spherical nanoparticles *via* RAFT dispersion polymerization of BzMA in mineral oil

$PLMA_{63}$-$PBzMA_{245}$ spherical nanoparticles were synthesized at 20% w/w solids as follows. $PLMA_{63}$ precursor (0.27 g; 16.56 µmol), BzMA (0.73 g; 4.14 mmol; target DP = 250), T21s initiator (1.19 mg; 5.52 µmol; precursor/T21s molar ratio = 3.0; 10.0% v/v in mineral oil) and mineral oil (4.00 g) were weighed into a glass vial and purged with nitrogen for 30 min. The sealed vial was immersed in a preheated oil bath at 90 °C and the reaction mixture was magnetically stirred for 6 h. ^{1}H NMR analysis indicated 98% conversion by comparing the integrated methylene signal at 5.25 ppm assigned to BzMA monomer to the integrated methylene signal for PBzMA at 4.80–5.10 ppm. THF GPC analysis indicated an M_n of 38 700 g mol^{-1} and an M_w/M_n of 1.18. A hydrodynamic diameter (D_h) of 48 nm (polydispersity index = 0.03) was determined by DLS.

Synthesis of poly(lauryl methacrylate-*stat*-glycidyl methacrylate)-poly(benzyl methacrylate) [P(LMA_{50}-*stat*-$GlyMA_9$)-$PBzMA_{245}$] spherical nanoparticles *via* RAFT dispersion polymerization of BzMA in mineral oil

P(LMA_{50}-*stat*-$GlyMA_9$)-$PBzMA_{245}$ spherical nanoparticles were synthesized at 20% w/w solids as follows. P(LMA_{50}-*stat*-$GlyMA_9$) precursor (0.25 g; 17.52 µmol), BzMA (0.77 g; 4.38

mmol; target DP = 250), T21s initiator (1.26 mg; 5.84 µmol; precursor/T21s molar ratio = 3.0; 10.0% v/v in mineral oil) and mineral oil (4.09 g) were weighed into a glass vial and purged with nitrogen for 30 min. The sealed vial was immersed in a preheated oil bath at 90 °C and the reaction mixture was magnetically stirred for 6 h. ^{1}H NMR analysis indicated 98% conversion by comparing the integrated methylene signal at 5.25 ppm assigned to BzMA monomer to the integrated methylene signal of PBzMA at 4.80–5.10 ppm. THF GPC analysis indicated an M_n of 39 500 g mol^{-1} and an M_w/M_n of 1.18. A hydrodynamic diameter (D_h) of 56 nm (polydispersity index = 0.04) was determined by DLS.

^{1}H NMR Spectroscopy

^{1}H NMR spectra were recorded in either CD_2Cl_2 or $CDCl_3$ using a 400 MHz Bruker Avance spectrometer. Typically, 64 scans were averaged per spectrum.

Gel Permeation Chromatography (GPC)

Molecular weight distributions were assessed by GPC using THF as an eluent. The GPC system was equipped with two 5 µm (30 cm) Mixed C columns and a WellChrom K-2301 refractive index detector operating at 950 ± 30 nm. The THF mobile phase contained 2.0% v/v triethylamine and 0.05% w/v butylhydroxytoluene (BHT) and the flow rate was fixed at 1.0 ml min^{-1}. A series of twelve near-monodisperse poly(methyl methacrylate) calibration standards (M_p values ranging from 800 to 2 200 000 g mol^{-1}) were used in combination with a refractive index detector.

Dynamic Light Scattering (DLS)

DLS studies were performed using a Zetasizer Nano ZS instrument (Malvern Instruments, UK) at a fixed scattering angle of 173°. Copolymer dispersions were diluted to 0.10% w/w solids using *n*-dodecane prior to analysis at 20 °C. The z-average diameter and DLS polydispersity were calculated by cumulants analysis of the experimental correlation function using Dispersion Technology Software version 6.20. Data were averaged over ten runs each of thirty seconds duration.

Transmission Electron Microscopy (TEM)

TEM studies were conducted using a FEI Tecnai G2 spirit instrument operating at 80 kV and equipped with a Gatan 1k CCD camera. A single droplet of a 0.10% w/w copolymer dispersion was placed onto a carbon-coated copper grid and allowed to dry, followed by exposure to ruthenium(VIII) oxide vapor for 7 min at 20 °C.[2] This heavy metal compound acts as a positive stain for the core-forming PGlyMA or PMMA block to improve contrast. The ruthenium(VIII) oxide was prepared as follows: ruthenium(IV) oxide (0.30 g) was added to water (50 g) to form a black slurry; addition of sodium periodate (2.0 g) with continuous stirring produced a yellow solution of ruthenium(VIII) oxide within 1 min at 20 °C.

Small-Angle X-ray Scattering (SAXS)

SAXS patterns were recorded using a Xeuss 2.0 laboratory beamline (Xenocs, Grenoble, France) equipped with a MetalJet X-ray source (Ga$K\alpha$ radiation, wavelength λ = 1.34 Å, with q ranging from 0.03 to 2.00 nm^{-1}, where $q = 4\pi.\sin\theta/\lambda$ is the length of the scattering vector and θ is one-half of the scattering angle) and a 2D Pilatus 1M pixel detector (Dectris, Baden-Daettwil, Switzerland). A glass capillary of 2.0 mm diameter was used as a sample holder. Scattering data were reduced using software supplied by the SAXS instrument manufacturer (Xenocs) and were further analyzed using Irena SAS macros for Igor Pro.[3] SAXS patterns were fitted by fixing the radius of gyration (R_g) for the $PLMA_{63}$ and P(LMA_{50}-*stat*-$GlyMA_9$) precursors at 2.70 nm and 2.61 nm, respectively.

Scanning Electron Microscopy (SEM)

SEM images were obtained using an FEI Inspect-F instrument operating at an accelerating voltage of 10 kV. Stainless steel substrates were dried and then sputter-coated with a thin overlayer of gold prior to imaging. The MTM disks were immersed in *n*-hexane to remove mineral oil before drying and subsequent sputter-coating with a thin gold overlayer.

Quartz Crystal Microbalance with Dissipation (QCM-D)

QCM-D measurements were performed using an openQCM NEXT instrument (Novaetech S.r.l., Pompeii, Italy) equipped with a temperature-controlled cell connected to a Masterflex Digital Miniflex peristaltic pump (Cole-Parmer Instrument Company, UK). The stainless steel substrates (SS2343, 5 MHz) were supplied by Q-Sense AB (Sweden). Prior to adsorption experiments, each substrate was thoroughly cleaned by sonication in DMF, ethanol and acetone in turn (for 10 min in each solvent), followed by exposure to UV/ozone for 10 min and finally dried with compressed air. The substrates were initially equilibrated with *n*-dodecane, followed by the introduction of the nanoparticle dispersion (1.0% w/w solids, prepared by dilution with *n*-dodecane) then equilibrated with *n*-dodecane again. Measurements were performed at either 20 or 40 °C at a constant flow rate of 0.5 mL min^{-1} to ensure that fresh solution was always present within the cell.

Lubrication Testing Using a Mini-Traction Machine (MTM)

The nanoparticle dispersion was diluted to 2.5% w/w solids using an API Group III mineral base oil at 20 °C (with no other additives present) then magnetically stirred while heating up to 65 °C, held for a further 5 min at 65 °C with stirring, then allowed to cool to 20 °C before addition to the mini-traction machine (MTM, PCS Instruments, UK). MTM was used to evaluate the lubricating performance of each nanoparticle dispersion in turn. The MTM ball and disk were each made from AISI 52100 steel, and had a surface roughness of less than 0.02 µm Ra. The disk had a mean diameter of 46 mm and the ball had a mean diameter of 19.05 mm. Stribeck curves were recorded for each nanoparticle dispersion at entrainment speeds ranging from 3000 to 30 mm s^{-1} with a slide-to-roll ratio (SRR) of 50% under constant load (37 N) at 40, 60 and 80 °C. The applied load was 37 N, which equates to a contact pressure of 1 GPa. Nanoparticle dispersions were evaluated at a constant load (37 N) and entrainment speed while increasing the temperature at a linear ramp rate (heating rate = 1.33 °C min^{-1}) from 40 to 120 °C. In this latter set of experiments, the entrainment speed was set at 200 mm s^{-1} with an SRR of 50%.

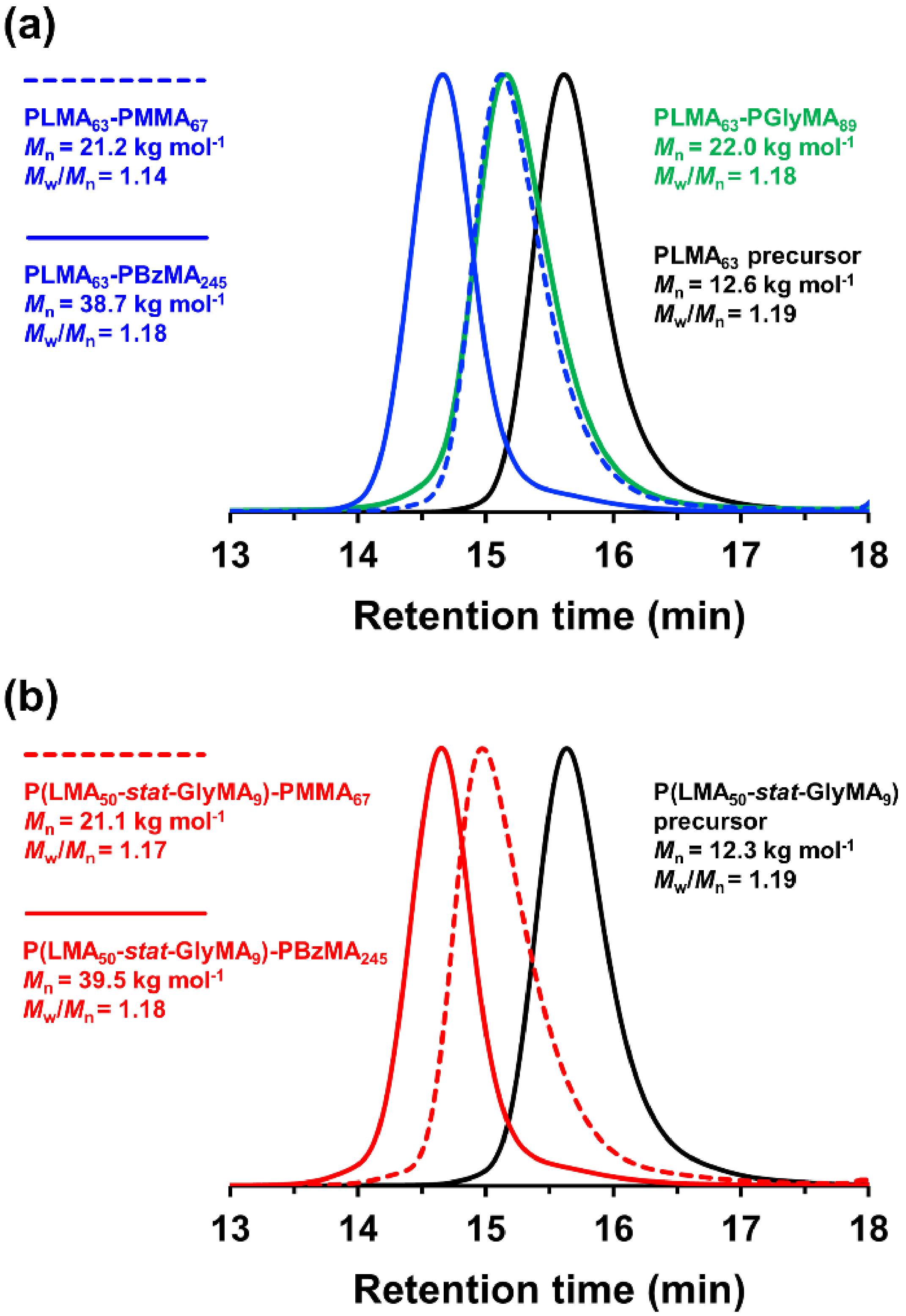

Figure S1. THF GPC curves (*vs.* a series of near-monodisperse poly(methyl methacrylate) calibration standards; refractive index detector) recorded for: **(a)** the $PLMA_{63}$ precursor and the corresponding $PLMA_{63}$-$PGlyMA_{89}$, $PLMA_{63}$-$PMMA_{67}$, and $PLMA_{63}$-$PBzMA_{245}$ diblock copolymers; **(b)** the P(LMA_{50}-*stat*-$GlyMA_9$) precursor and the corresponding P(LMA_{50}-*stat*-$GlyMA_9$)-$PMMA_{67}$ and P(LMA_{50}-*stat*-$GlyMA_9$)-$PBzMA_{245}$ diblock copolymers.

Table S1. Summary of the structural parameters obtained by fitting SAXS patterns for the $PLMA_{63}$-$PMMA_{67}$, $PLMA_{63}$-$GlyMA_{89}$, P(LMA_{50}-*stat*-$GlyMA_9$)-$PMMA_{67}$, $PLMA_{63}$-$PBzMA_{245}$ and P(LMA_{50}-*stat*-$GlyMA_9$)-$PBzMA_{245}$ nanoparticles using a spherical micelle model.[4] D_v is the overall volume-average sphere diameter such that $D_v = D_c + 4R_g$, where D_c is the mean core diameter and R_g is the radius of gyration of the stabilizer chains. N_{agg} is the mean aggregation number. The corresponding hydrodynamic diameter (D_h) and polydispersity (PDI) reported by DLS are also included.

Copolymer Composition	SAXS			DLS	
	D_c (nm)	D_v (nm)	N_{agg}	D_h (nm)	PDI
$PLMA_{63}$-$PMMA_{67}$	10.8 ± 0.4	21.6 ± 0.4	140	27	0.05
$PLMA_{63}$-$PGlyMA_{89}$	12.2 ± 0.2	23.0 ± 0.2	130	28	0.03
P(LMA_{50}-*stat*-$GlyMA_9$)-$PMMA_{67}$	11.2 ± 0.4	21.6 ± 0.4	150	26	0.05
$PLMA_{63}$-$PBzMA_{245}$	29.8 ± 2.7	40.6 ± 2.7	390	48	0.03
P(LMA_{50}-*stat*-$GlyMA_9$)-$PBzMA_{245}$	36.4 ± 3.9	46.8 ± 3.9	620	56	0.04

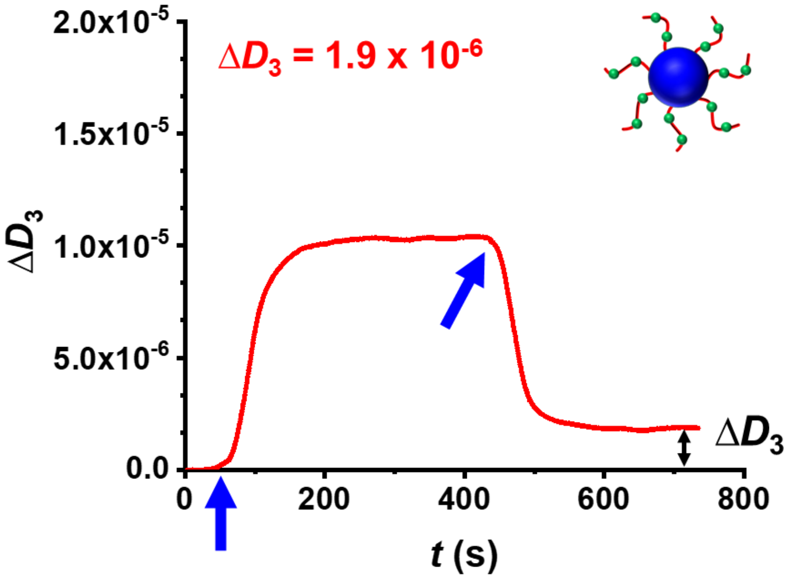

Figure S2. The change in dissipation (ΔD_3) recorded during the adsorption of a 1.0% w/w dispersion of P(LMA_{50}-*stat*-$GlyMA_9$)-$PMMA_{67}$ nanoparticles from *n*-dodecane onto a stainless steel substrate at a flow rate of 0.50 mL min^{-1} at 20 °C. The curve is shown for a single measurement but it is representative of the average of two experiments. Blue arrows indicate (i) the introduction of the nanoparticles into the QCM cell and (ii) the rinsing step with pure *n*-dodecane. The black double-headed arrow indicates the final change in dissipation (ΔD_3).

Table S2. Summary of Δf_3, ΔD_3, $\Delta D_3/(-\Delta f_3/3)$, Γ, Γ_t and Θ data obtained from the QCM-D analysis of the surface adsorption of ~27 nm $PLMA_{63}$-$PMMA_{67}$, $PLMA_{63}$-$PGlyMA_{89}$ and P(LMA_{50}-*stat*-$GlyMA_9$)-$PMMA_{67}$ nanoparticles onto a model stainless steel substrate. Γ was calculated from Δf_3 using the Sauerbrey equation (**Equation 1**). Γ_t was calculated using **Equation 2**. Θ was obtained by dividing Γ by Γ_t.

Nanoparticle Dispersion	T (°C)	Δf_3 (Hz)	Mean Δf_3 (Hz)	Mean Γ (mg m^{-2})	Γ_t (mg)	Θ	ΔD_3 (10^{-6})	Mean ΔD_3 (10^{-6})	$\Delta D_3/(-\Delta f_3/3)$ (10^{-8})
**$PLMA_{63}$-$PMMA_{67}$** 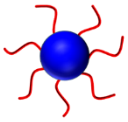	20	-63	-65	3.8	31.9	0.12	1.1	1.1	5.2
	20	-67					1.2		
	40	-42	-43	2.5	31.9	0.08	0.2	0.2	1.3
	40	-44					0.1		
**$PLMA_{63}$-$PGlyMA_{89}$** 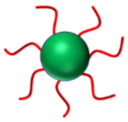	20	-65	-63	3.7	28.9	0.13	0.2	0.3	1.7
	20	-61					0.5		
	40	-44	-45	2.7	28.9	0.09	0.3	0.3	2.0
	40	-46					0.3		
**P(LMA_{50}-*stat*-$GlyMA_9$)-$PMMA_{67}$** 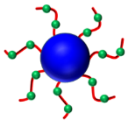	20	-126	-129	7.6	31.9	0.24	1.7	1.8	4.2
	20	-131					1.9		
	40	-147	-151	8.9	31.9	0.28	1.0	1.6	3.2
	40	-154					2.1		

Table S3. Summary of Δf_3, ΔD_3, $\Delta D_3/(-\Delta f_3/3)$, Γ, Γ_t and Θ data obtained from the QCM-D analysis of the surface adsorption of ~50 nm $PLMA_{63}$-$PBzMA_{245}$ and P(LMA_{50}-*stat*-$GlyMA_9$)-$PBzMA_{245}$ nanoparticles onto a model stainless steel substrate. Γ was calculated from Δf_3 using the Sauerbrey equation (**Equation 1**). Γ_t was calculated using **Equation 2**. Θ was obtained by dividing Γ by Γ_t.

Nanoparticle Dispersion	T (°C)	Δf_3 (Hz)	Mean Δf_3 (Hz)	Mean Γ (mg m^{-2})	Γ_t (mg)	Θ	ΔD_3 (10^{-6})	Mean ΔD_3 (10^{-6})	$\Delta D_3/(-\Delta f_3/3)$ (10^{-8})
**$PLMA_{63}$-$PBzMA_{245}$** 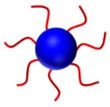	20	-110	-109	6.4	59.0	0.11	1.1	1.9	5.2
	20	-107					2.6		
**P(LMA_{50}-*stat*-$GlyMA_9$)-$PBzMA_{245}$** 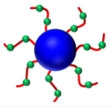	20	-524	-530	31.3	59.0	0.53	2.9	3.1	1.8
	20	-536					3.3		

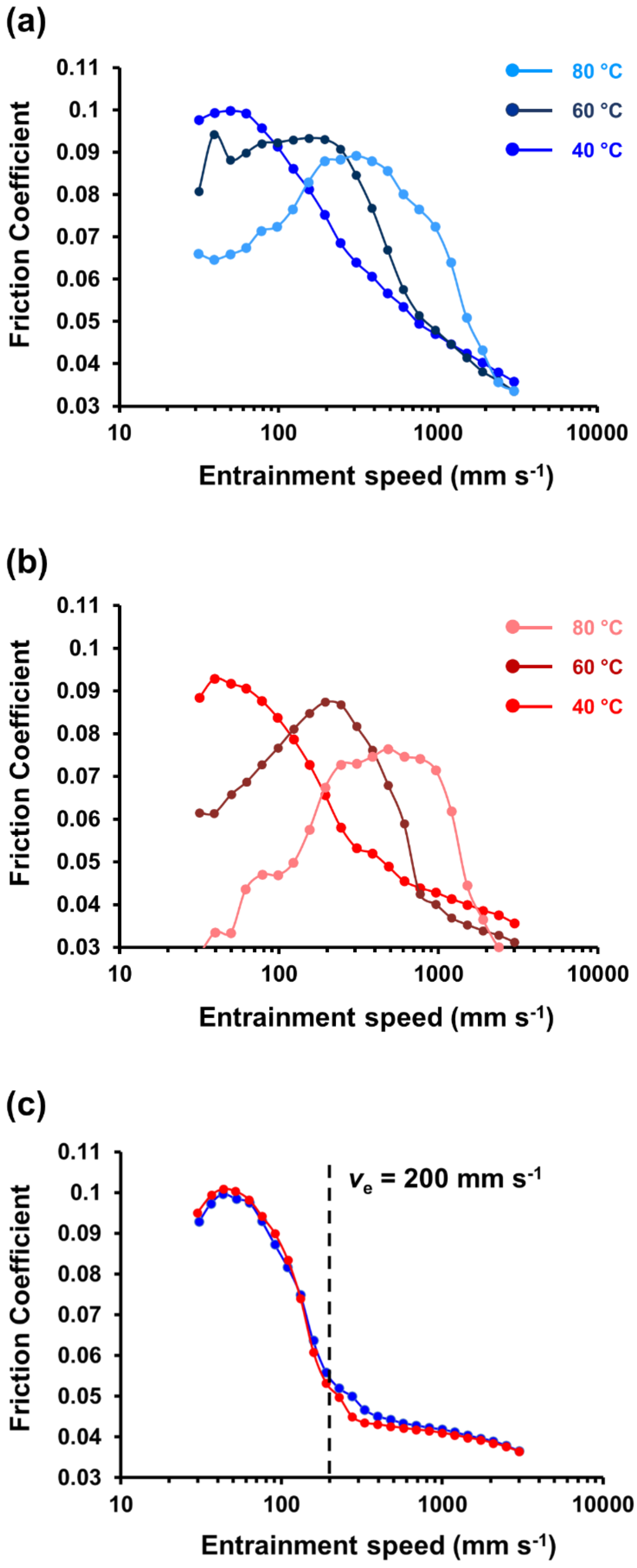

Figure S3. Stribeck curves showing the change in friction coefficient with entrainment speed for 2.5% w/w dispersions of **(a)** $PLMA_{63}$-$PBzMA_{245}$ and **(b)** P(LMA_{50}-*stat*-$GlyMA_{9}$)-$PBzMA_{245}$ nanoparticles at 40, 60 and 80 °C. **(c)** Comparison of the Stribeck curves obtained for $PLMA_{63}$-$PBzMA_{245}$ (blue data) and P(LMA_{50}-*stat*-$GlyMA_{9}$)-$PBzMA_{245}$ (red data) nanoparticles at 40 °C. Data were recorded at a 50% slide-to-roll ratio (SRR) under an applied load of 37 N.

References

1 C. György, T. Smith, D. J. Growney and S. P. Armes, *Polym. Chem.*, 2022, **13**, 3619–3630.
2 J. S. Trent, *Macromolecules*, 1984, **17**, 2930–2931.
3 J. Ilavsky and P. R. Jemian, *J. Appl. Crystallogr.*, 2009, **42**, 347–353.
4 J. S. Pedersen, *J. Appl. Crystallogr.*, 2000, **33**, 637–640.
